# Summer diapause induced by high temperatures in the oriental tobacco budworm: ecological adaptation to hot summers

**DOI:** 10.1038/srep27443

**Published:** 2016-06-07

**Authors:** Zhudong Liu, Yucui Xin, Yanan Zhang, Jianting Fan, Jianghua Sun

**Affiliations:** 1 The State Key Laboratory of Integrated Management of Pest Insects and Rodents, Institute of Zoology, Chinese Academy of Sciences, Beijing 100080, China; 2 Institute of Health Sciences, Anhui University, Hefei 230039, China; 3 College of Forests and Biology, Agriculture and Forest University of Zhejiang, Hangzhou 310000, China

## Abstract

Summer diapause in *Helicoverpa assulta* (Hübner), which prolongs the pupal stage, particularly in males, is induced by high temperatures. In the laboratory, 3^rd^-, 4^th^-, 6^th^-instar and prepupal larvae were exposed to high temperatures – 33 and 35 °C with a photoperiod of LD16:8 – until pupation to induce summer diapause. The results showed that the incidence of summer diapause was influenced by temperature, stage exposed, and sex. The higher the temperature, the more often summer diapause was attained. Sixth-instar and prepupal larvae were the sensitive stages for summer diapause induction. *H. assulta* summer-diapausing pupae needed diapause development to resume development when temperatures became favorable. Furthermore, both body mass and energy storage capacity (lipid and glycogen) were significantly affected by diapause rather than sex, and were significantly higher in summer-diapausing pupae than in non-diapausing pupae. In addition, the body mass loss and respiration rate showed that the rate of metabolism in the summer-diapausing pupae was consistently lower than in non-diapausing pupae, which were significantly affected by diapause and pupal age. We conclude that summer diapause in *H. assulta* is a true diapause, and *H. assulta* has evolved mechanisms to accumulate energy storage and to lower its metabolism to adapt to hot summers.

The oriental tobacco budworm *Helicoverpa assulta*, a sister species of the cotton bollworm *H. armigera*, occurs sympatrically with *H. armigera* and is a serious crop pest in China and other areas of eastern Asia^1^,^2^. These two sister species appear similar and have similar feeding behaviors, but their host-plant ranges are quite different. *H. armigera* is a highly polyphagous herbivore; its host plant range includes at least 60 crop species, such as cotton, corn, wheat, soybean, tobacco and tomato, and 67 wild plant species from about 30 plant families, including Malvaceae, Solanaceae, Gramineae, and Leguminosae^3^,^4^,^5^,^6^. However, *H. assulta* is an oligophagous species with a relatively narrow host-plant range, and mainly specializes on Solanaceae, such as tobacco, hot pepper, and several *Physalis* species^1^,^3^,^7^. The two sister species overlap from middle May to middle October and both over-winter with diapausing pupae^7^,^8^,^9^,^10^. Even since Wang and Dong^11^ found that the interspecific hybridization of these two sister species is feasible, they have been used as a model to explore the genetic basis for speciation^12^,^13^. However, although diapause in *H. armigera*, including both winter and summer diapause, is well known^8^,^9^,^10^,^14^,^15^,^16^, no research has been done on the summer diapause of its sister species, the oriental tobacco budworm. The lack of information about *H. assulta* has hampered our understanding of how the insects deal with hot summers and the comparison study using the two sibling species to understand the mechanism of summer diapause.

Summer diapause (aestivation) is a developmental resting stage that insects use to avoid hyperthermia and other associated physiological stresses (dehydration, starvation)^17^. It is also an important mechanism for synchronizing life cycles with the growing season^18^,^19^. Because of climate change and the resulting increased incidence of heat stress, more attention is currently being paid to heat stress on ectotherms^20^. High temperatures are known to induce summer diapause in *H. virescens* (F.) in Arizona^21^, and a similar phenomenon has also been observed for *H. punctigera* in the Namoi Vally and *H. armigera* in the Ord of Australia^22^. More detailed evidence for summer diapause in *H. armigera* was lacking until Nibouche^14^ described a “hot thermal diapause” initiated at 37 °C in a population from Burkina Faso. In China, summer diapause in *H. armigera*, induced by high temperatures, has been also demonstrated^15^. However, the performance of its sister species *H. assulta* at high temperatures has not been studied. Does *H. assulta* have the same ability to enter summer diapause?

The term “diapause” has been used for developmental delays that allow organisms to survive unfavorable conditions, and to synchronize growth and development with favorable conditions^23^. It is important to distinguish between diapause, in which development is arrested in advance of unfavorable conditions and does not respond immediately to the amelioration of the external environment, and quiescence, in which development is temporarily inhibited by an unfavorable environment and may be resumed as soon as the hindrance is removed^24^. During diapause, there is a refractory phase^25^, also known as “diapause development”^24^. The development is inhibited even if environmental conditions have become favorable again. In addition, diapause stimuli are perceived before the induction of diapause begins, usually during a stage prior to the one in which the insect enters diapause^23^. Moreover, the diapausing insect has some physiological preparedness, such as accumulated energy storage capacity and decreased metabolism, as pointed out by Tombes^26^.

The adaptive significance of winter diapause in *H. assulta* is obvious: it allows pupae to survive otherwise lethal winter temperatures^27^. However, whether *H. assulta* experiences summer diapause and the adaptive significance of summer diapause have until now remained unclear. The host-plants of the pest are usually available in summer, and the main environmental factor that is known to induce summer diapause is high temperatures. Summers are becoming even hotter with climate change and will strongly affect animal species at middle latitudes^20^,^28^,^29^. In many species of moths, including the cotton bollworm, *Helicoverpa armigera*, males tend to produce malformed spermatozoa when moths are exposed to high temperatures^30^,^31^. Pupal diapause postpones the emergence of adults until conditions are cooler. In most temperate parts of China, the hottest times are late July and early August: daily maximum temperatures may reach 40 °C, and the temperature of the soil surface may be much higher^15^. To avoid hyperthermia and the rapid depletion of energy reserves as a result of such deleterious high temperatures, the oriental tobacco budworm may enter summer diapause. How prepared is it to over hot summer?

## Results

### Effects of high temperatures on the incidence of summer diapause and the diapause-sensitive stage

**Figure 1:**
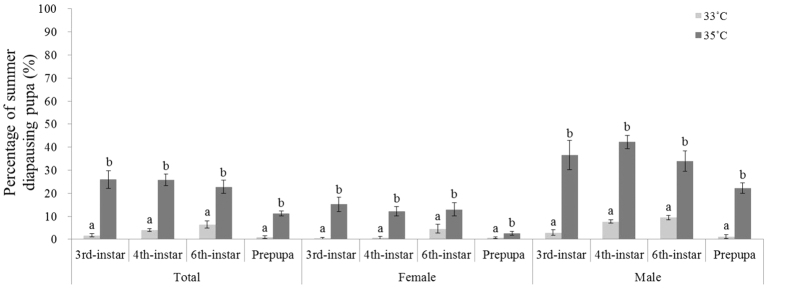
The incidence of summer diapause among larvae after 3^rd^-, 4^th^-, 6^th^-instar and prepupal larvae were exposed to temperatures 33 and 35 °C. The bar indicates Mean ± SE, and different letters indicate significant differences between the two tested temperatures at *p* < 0.05 by the generalized linear model.

The results showed that the incidence of diapause was significantly affected by temperatures, stage exposed, and sex (temperature, Wald Chi-Square = 245.546, df = 1, *p* < 0.0001; stage, Wald Chi-Square = 42.089, df = 3, *p* < 0.0001; sex, Wald Chi-Square = 136,265, df = 1, *p* < 0.0001; temperature × stage, Wald Chi-Square = 21.349, df = 3, *p* < 0.0001; temperature × sex, Wald Chi-Square = 63.643, df = 1, *p* < 0.001; stage × sex, Wald Chi-Square = 7.214, df = 3, *p* = 0.065; temperature × stage × sex, Wald Chi-Square = 1.891, df = 3, *p* = 0.595). Fewer of *H. assulta* individuals, on average, enter summer diapause under 33 °C with L:D = 16:8, and this figure increased to more than 20% of individuals under 35 °C with L:D = 16:8 (Fig. 1). Moreover, the incidence of diapause among both females and males significantly increased at all tested stages except for the females’ prepupal stage when insects were exposed to higher temperature 35 °C compared with 33 °C (Fig. 1).

**Figure 2:**
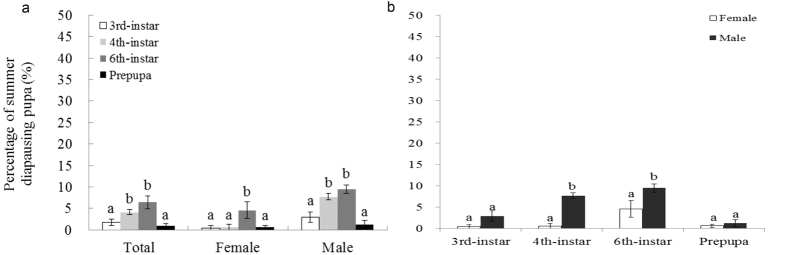
The incidence of summer diapause between stages exposed to temperature 33 °C (**a**) and sexes (**b**). The bar indicates Mean ± SE, and different letters indicate significant differences between the two tested temperatures at *p* < 0.05 by the generalized linear model.

**Figure 3:**
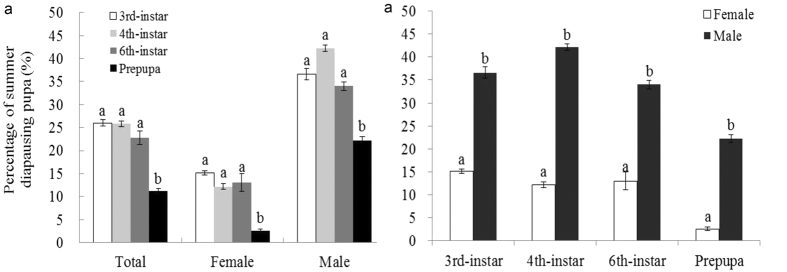
The incidence of summer diapause among larvae at stages exposed to temperature 35 °C (**a**) and sexes (**b**). The bar indicates Mean ± SE, and different letters indicate significant differences between the two tested temperatures at *p* < 0.05 by the generalized linear model.

The temperature of 33 °C could trigger summer diapause in *H. assulta*, and the incidence of diapause was significantly affected by stage and sex (stage, Wald Chi-Square = 59.511, df = 3, *p* < 0.0001; sex, Wald Chi-Square = 39.651, df = 1, *p* < 0.0001; stage × sex, Wald Chi-Square = 17.456, df = 3, *p* = 0.001). At 33 °C, the incidence of diapause was significantly different among stages exposed; the highest incidence was attained for females and males when 6^th^-larvae and 4^th^-larvae were exposed to high temperature until pupation, respectively (Fig. 2a). Moreover, male insects fell significantly more easily into diapause than did female insects when 4^th^, and 6^th^-instar larvae were exposed to the high temperature (Fig. 2b). At 35 °C, the incidence of diapause was significantly affected by stage and sex (stage, Wald Chi-Square = 28.785, df = 3, *p* < 0.0001; sex, Wald Chi-Square = 111.627, df = 1, *p* < 0.0001; stage × sex, Wald Chi-Square = 3.541, df = 3, *p* = 0.316). The incidence of diapause among larvae at the 3^rd^, 4^th^, and 6^th^ instars when treated at the high temperature (35 °C) was significantly higher than that of prepupal larvae exposed to the high temperature (Fig. 3a). Moreover, male insects fell significantly more easily into diapause at each tested stage than did female insects (Fig. 3b).

**Figure 4:**
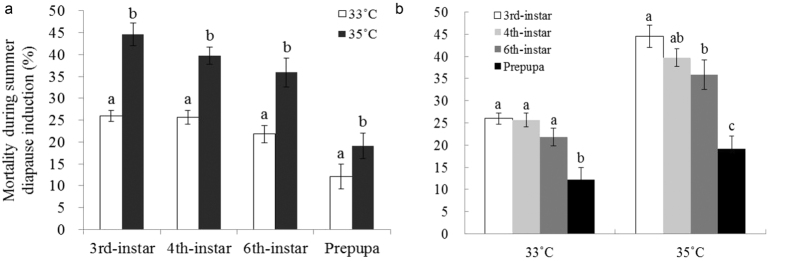
The comparison of mortality during summer diapause induction among stages at 33 and 35 °C. (**a**) the comparison of mortality between 33 and 35 °C; (**b**) the comparison of mortality between stages exposed to 33 and 35 °C. The bar indicates Mean ± SE and different letters indicate significant differences between at p < 0.05 by the generalized linear model.

Meanwhile, the results showed that mortality during summer diapause induction was significantly affected by both temperature and stage exposed (temperature, Wald Chi-Square = 79.366, df = 1, *p* < 0.0001; stage, Wald Chi-Square = 100.446, df = 3, *p* < 0.0001; temperature × stage, Wald Chi-Square = 7.667, df = 3, *p* = 0.053). The higher the temperature, the higher the mortality (Fig. 4a). Moreover, 3^rd^, 4^th^, and 6^th^ larvae had higher mortality when were exposed to the high temperature than did prepupae (Fig. 4b).

### Comparison of retention time of eye spots

As shown in Table 1A, average retention times of summer-diapausing pupae, non-diapausing pupae reared at 35 °C and non-diapausing pupae reared at 27 °C were 13.4, 1.8, and 1.6 d, respectively. Retention times of eye spots were significantly longer among summer-diapausing pupae than among non-diapausing pupae at 35 °C or non-diapausing pupae reared at 27 °C (Wald Chi-Square = 293.759, df = 2, *p* < 0.0001).

When summer-diapausing pupae were kept 5 d at 35 °C, and then transferred to 30 °C and 27 °C to terminate diapause, the retention times was 8.5 d, which was significantly longer than the retention time of non-diapausing pupae reared at 27 and 35 °C (Table 1B) (Wald Chi-Square = 145.842, df = 2, *p* < 0.0001).

### Body mass and water content

Body mass were recorded starting the second day after pupation (Table 2). The results showed that body mass was affected by the status of diapause rather than by sex (diapause, F = 10.807, df = 1, 221, *p* = 0.001; sex, F = 1.173, df = 1, 221, *p* = 0.280; diapause × sex, F = 0.315, df = 1, 221, *p* = 0.575). The body mass of summer-diapausing pupae (268.0 mg) was significantly heavier than that of non-diapausing pupae (247.2 mg) (ANOVA: F = 12.231; df = 1, 223; *p* = 0.01). However, water content was not affected by neither diapause nor sex (diapause, F = 0.062, df = 1, 52, *p* = 0.804; sex, F = 0.563, df = 1, 52, *p* = 0.456; diapause × sex, F = 1.909, df = 1, 52, *p* = 0.173). The water content was 63.5% and 63.2%, respectively, for diapausing and non-diapausing pupae reared at 35 °C (ANOVA: F = 0.637; df = 1, 54; *p* = 0.428).

### Energy storage

Comparisons of lipid and glycogen levels are shown in Table 2. With regard to absolute amounts, mg/pupa, the result showed that lipid and glycogen were significantly affected by diapause (lipid: diapause, F = 13.051, df = 1, 38, *p* = 0.001; sex, F = 3.128, df = 1, 38, *p* = 0.085; diapause × sex, F = 0.028, df = 1, 38, *p* = 0.868. Glycogen: diapause, F = 5.909, df = 1, 8, *p* = 0.041; sex, F = 0.145, df = 1, 8, *p* = 0.713; diapause × sex, F = 0.037, df = 1, 8, *p* = 0.853). Diapausing pupae contained significantly more lipid and glycogen levels than did non-diapausing pupae (Table 2) (lipids: 40.3 and 48.5 mg/pupa for non-diapausing pupae and summer-diapausing pupae reared at 35 °C, respectively (ANOVA: F = 9.992; df = 1, 40; *p* = 0.003); glycogen: 4.1 and 2.3 mg/pupa for summer-diapausing pupae and non-diapausing pupae reared at 35 °C, respectively (ANOVA: F = 7.865; df = 1, 10; *p* = 0.019)). However, as measured (mg/g DW), relative lipid content was not, but relative glycogen content was, significantly affected by diapause (lipid: diapause, F = 0.002, df = 1, 38, *p* = 0.963; sex, F = 0.415, df = 1, 38, *p* = 0.523; diapause × sex, F = 0.040, df = 1, 38, *p* = 0.842. Glycogen: diapause, F = 5.218, df = 1, 8, *p* = 0.050; sex, F = 0.645, df = 1, 8, *p* = 0.445; diapause × sex, F = 0.024, df = 1, 8, *p* = 0.882).

### Dynamics of mass loss

**Figure 5:**
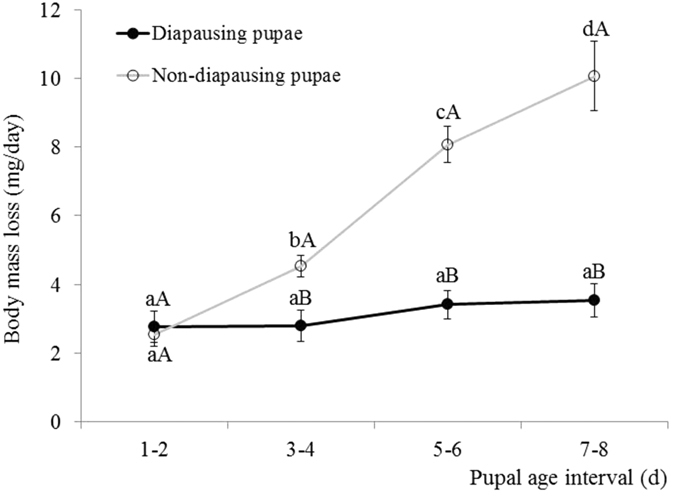
Curves of mass loss of *H. assulta* between diapausing and non-diapausing pupae at various pupal stages at 2-day interval. The bar indicates Mean ± SE. Different lower-case letters on the bars indicate significant differences between pupal age for each diapausing and non-diapausing pupa at p < 0.05. Different capital letters on the bars indicate significant differences between diapausing and non-diapausing pupae at p < 0.05.

The results showed mass loss was significantly affected by pupal age interval and diapause (pupal age interval, F = 26.718; df = 3, 321, *p* < 0.0001; diapause, F = 72.380, df = 1, 107, *p* < 0.0001; pupal age × diapause, F = 16.884, df = 3, 321, *p* < 0.0001). The mass loss curve for diapausing pupae showed that the amount of mass lost per day remained at low levels and did not change significantly with pupal age (ANOVA: F = 0.690; df = 3, 105; *p* = 0.560). However, the mass loss curve for non-diapausing pupae increased steeply with pupal age (ANOVA: F = 33.006; df = 3, 105; *p* < 0.001) (Fig. 5). Moreover, the mass loss of diapausing pupa was significantly lower than that of non-diapausing pupa reared at 35 °C except in the first two days (Fig. 4) (ANOVA: 1–2 day: F = 0.175; df = 1, 107; *p* = 0.676; 3–4 day: F = 10.59; df = 1, 107; *p* = 0.002; 5–6 day: F = 49.166; df = 1, 107; *p* < 0.001; 7–8 day: F = 39.115; df = 1, 107; *p* < 0.001).

### Respiration rate

**Figure 6:**
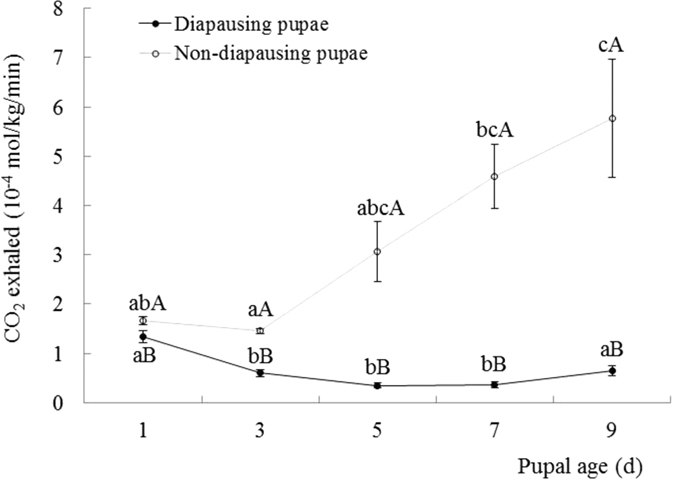
Dynamics of respiration rate between diapausing and non-diapausing pupae at various pupal ages. The bar indicates Mean ± SE. Different lower-case letters on the bars indicate significant differences between pupal age for each diapausing and non-diapausing pupa at p < 0.05. Different capital letters on the bars indicate significant differences between diapausing and non-diapausing pupae at p < 0.05.

The results showed respiration rate was significantly affected by pupal age and diapause (pupal age, F = 6.317; df = 4, 392, *p* < 0.0001; diapause, F = 84.781, df = 1, 98, *p* < 0.0001; pupal age × diapause, F = 9.158, df = 4, 392, *p* < 0.0001). The dynamics of respiration rates among diapausing and non-diapausing pupae reared at 1, 3, 5, 7, and 9 day old, measured as CO_2_ exhaled, are shown in Fig. 5. The respiration rate of diapausing pupae was rather flat, although the respiration rate at 1 day old was significantly higher than that of older diapausing pupae (ANOVA: F = 11.183; df = 4, 95; p < 0.001). However, for non-diapausing pupae, the respiration rate significantly increased with pupal age (ANOVA: F = 22.487; df = 4, 95; p < 0.001). Moreover, the respiration rate of diapausing pupae was significantly lower than that of non-dapausing pupae reared at any tested pupal age (Fig. 6) (ANOVA: 1^st^ day: F = 4.654; df = 1, 98; *p* = 0.033; 3^rd^ day: F = 94.772; df = 1, 98; *p* < 0.001; 5^th^ day: F = 19.882; df = 1, 98; *p* < 0.001; 7^th^ day: F = 42.099; df = 1, 98; *p* < 0.001; 9^th^ day: F = 17.880; df = 1, 98; *p* = 0.002).

## Discussion

Summer diapause may be defined as a diapause that is induced before the height of summer, and that is terminated and followed by reproductive, developmental, or feeding activities in autumn or winter^23^. Here, we provide evidence for the occurrence of summer diapause in *H. assulta* induced by high temperature. First, we reared larvae at different stages (3^rd^-, 4^th^-, 6^th^-instar and prepupal larvae) under high temperatures and found various percentages of pupae enter diapause. The incidence of summer diapause was influenced by both the temperature and the stage exposed. 3^rd^- and 4^th^-instar larvae exposed to a high temperature until pupation can’t significantly increase diapause incidence any more than 6^th^-instar larvae can, showing *H. assulta* final-instar and prepupal larvae were sensitive to the stimulus of high temperature. Moreover, males was more likely to enter summer diapause than were females. Comparing the retention times of eye spots, summer-diapausing pupae needed extra time to resume developing when temperatures became favorable. Moreover, both body mass and energy storage capacity (lipid and glycogen) were significantly higher in summer-diapausing pupae than in non-diapausing pupae, indicating *H. assulta* could accumulate energy storage before diapause. Furthermore, during diapause, *H. assulta* was able to decrease its mass loss and respiration to maintain a low metabolism to over a hot summer.

Sometimes it is difficult to discriminate summer diapause from “hot quiescence” or “hot thermal diapause” since the duration of summer diapause is normal very brief compared with the duration of winter diapause. Evidence from Nibouche^14^ and from our earlier work^15^ shows a true summer diapause occurs in *H. armigera*, in terms of anticipation of the induction, diapause development, energy storage capacity and metabolism. Similarly, we believe summer diapause in *H. assulta* is a true diapause and not a quiescence for the following reasons: First, diapause-stimuli are perceived before the stage of diapause begins, usually the stage prior to the one that enters diapause^23^,^32^. In *H. assulta*, as our current study shows, larvae in final-instar and prepual are sensitive to perceive stimulus, and diapause occurs at the pupal stage. Second, during diapause, there is a refractory phase^25^, also known as diapause development^24^. We did find that *H. assulta* summer-diapausing pupae had diapause development before they can resume development: *H. assulta* summer-diapausing pupae 8.5 days (at 30 °C) to finish diapause development, while the eye spots of non-diapausing pupae usually disappeared within 2 days. Third, our study showed that summer diapause pupa had the characteristics of accumulated energy storage capacity and low metabolism, which are characteristics shared with diapausing insects^26^.

As shown at 33 °C, the incidence of summer diapause was low and did not show the same trend as the incidence seen at 35 °C. We think 33 °C probably was the critical temperature for summer diapause induction; this would be similar to its sister species *H. armigera*, for which the critical temperature is 33 °C^15^. When the temperature was increased to 35 °C, the incidence of diapause was significantly increased compared with that at 33 °C. We also observed that only some individuals of this species enter summer diapause at 35 °C and that males were more likely than females to enter diapause at high temperatures. This sexual discrepancy confirms what has been previously reported^15^,^21^,^33^. In *H. armigera*, males are also more likely than females (63% compared to 11%) to enter summer diapause when temperatures are at 33–39 °C^15^; recall the earlier mention of the fact that males tended to produce malformed spermatozoa when moths were exposed to high temperatures^30^,^31^. Summer diapause may be the strategy *Helicoverpa* males have evolved to avoid damage from high temperatures by stopping pupal development and thereby escaping the adverse effects caused by high increasing temperatures.

Tombes^26^ pointed out that summer-diapausing insects shared four characteristics: low water content, low respiratory rates, high lipid levels, and undeveloped reproductive organs. We compared some characteristics related to metabolism between summer-diapausing and non-diapausing pupae: body mass, body mass loss per 2-day, lipid and glycogen levels and metabolism dynamics. In *H. assulta*, diapausing pupae were heavier than non-diapausing pupae and had the ability to store more energy, such as lipid and glycogen. However, no significant differences were found in water amounts between summer-diapausing and non-diapausing pupae. Low metabolism is one of the indicators of diapause, and we measured two indices to monitor metabolism, i.e., body mass loss and respiration rate, measured as the amount of CO_2_ exhaled. Body mass loss per 2-day in summer-diapausing pupae was very small, suggesting that metabolism was low. Similarly, the respiration rate of diapausing pupae resembled the trend with mass loss: diapausing pupae exhaled very low level of CO_2_ and their respiration curves were very flat. However, metabolism rates for non-diapausing pupae were high for both mass loss and respiration, and these increased linearly with pupal age. Low metabolism also characterizes the summer diapause of *Cymbalophora pudica*^34^ and *H. armigera*^15^. Like its sister species *H. armigera*^15^, *H. assulta* seems to have evolved summer diapause to accumulate energy storage capacity and to decrease its metabolism over a hot summer.

Summer diapause is not as intense as winter diapause in *H. assulta*. Winter-diapausing pupae needed about 300 days to finish diapause development at 22 °C (Liu unpublished data), while summer-diapausing pupae needed only 13 days to terminate diapause at high temperatures. Furthermore, when summer-diapausing pupae kept 5 days at 35 °C were transferred to 30 °C to terminate diapause, they took 8.5 d to resume development, indicating summer diapause is more flexible than winter diapause. Winter diapause in *H. assulta* is a strategy to withstand cold winters, and is induced by low temperatures and, during pre-winter, a short photoperiod^27^; very low metabolism rates during the long cold winter are the result^35^,^36^. Winter is long (about 4 months), whereas the hot summer is short (about two weeks), which may be why winter diapause lasts much longer than summer diapause. In summer, *H. assulta* final-instar larvae develop mainly on the top of agricultural crops exposed to hot, dry conditions, and prepupae pupate under shallow soil. In areas where *H. assulta* is found, mainly the south of China, temperatures in late July and August are very high, sometimes reaching 40 °C, and ground temperature is even higher; such temperatures may induce *H. assulta* to enter into summer diapause. Moreover, climate change will increase both average temperatures and extreme summer temperatures, which will strongly threaten ectotherms at middle latitudes^20^,^28^,^29^. In response to climate warming, some insects increase numbers of generations^37^,^38^,^39^, spread to wider distributions^40^,^41^. On the other hand, climate change may affect the initiation of diapause^37^. Whether summer diapause in *H. assulta* will be shaped by climate warming is remained unknown. In addition, summer diapause of *H. assulta* has not been identified in the field; three factors make it difficult to observe in the field: its phenotypic plasticity in response to summer diapause, relatively short diapause development, and the fact that it pupate in soil. However, we did find a report that showed the pupal stage of *H. assulta* lasted about 17 days in August when it is in hot summer in Hunan Province^42^– roughly 7 days longer than the pupal stage of other generaions– which may be due to summer diapause.

We believe that *H. assulta* has evolved this strategy to deal with summers that are extremely hot. Summer diapause in *H. assulta* is maintained by high temperatures during summer and can be rapidly ended by moderate temperatures; typically this is how larvae adapt to short hot summers. Moreover, *H. assulta* has evolved physiological mechanisms to accumulate energy storage capacity and maintain its low metabolism in response to high temperature. By entering into summer diapause, first, *H. assulta* may extend its pupal stage to delay its adult emergence and thereby avoid producing malformed spermatozoa caused by potential high temperatures^30^,^31^; this is seen in *Heliothis virescens*^21^. Summer diapause in *H. assulta* is rapidly ended by comfortable temperatures; as a result, the emergence of adults from diapausing pupae is delayed about two weeks to synchronize with favorable temperatures of summer. This synchronization serves as a negative feedback system to stabilize the species’ seasonal life cycle. Meanwhile, in *H. assulta*, a portion (about 30% of pupae reared at 35 °C and a higher percentage of those reared at higher temperatures) are dormant during periods of high temperatures, but the rest continue to develop. Like other types of polymodel emergence strategies, which have been described as “bet hedging”^43^, this may protect the species from unpredictable risks due to a fluctuating environment.

## Materials and Methods

### Establishing a laboratory colony

The population of *H. assulta* was established with larvae collected on tobacco growing in Xiangxi Automonous County, Hunan province, China, in 2009. The collected larvae were then kept in individual glass tubes (2.0 cm dia. × 8.0 cm high), reared on an artificial diet^44^ to minimize the possible experience of host plants at this stage^45^. The larvae were reared at 27 °C under a photoperiod of L:D 14:10 h to prevent winter diapause and were allowed to pupate in the glass tubes. Pairs of eclosed adults were allowed to mate in plastic mating cups (10 cm diameter × 8 cm high) and provided with 10% honey solution. Eggs were collected, and the larvae of the new generation were put on a fresh artificial diet in Petri dishes (10 cm diameter × 2.0 cm high). Newly hatched larvae were reared in groups in the Petri dishes with diet until the 3^rd^ instar, after which they were separated and placed in individual glass tubes to prevent cannibalism^46^. *H. assulta* colonies had been maintained for more than 6 generations in the laboratory before the start of the experiments to reduce the possible influence of the host source^47^.

### Criteria for summer diapause

Two characteristics, eye spots and fat body, were used to determine whether pupae were in diapause (Supplementary Information Fig. S1). The first was the retention of pigmented eye spots in the postgenal region^48^. Like the temperatures that brought about summer diapause in *H. armigera*^15^, high temperatures (35 °C) induced summer diapause in *H. assulta*, and eye spots of non-diapausing pupa moved and disappeared during the first two days after pupation^49^. In this study, pupae showing eye spots for five days after pupation at a high temperature (33 and 35 °C) were considered to be diapausing. The second characteristic that was deemed to constitute diapause was the condition of the fat body in the pupal abdomen^50^. The fat body of newly formed pupae is composed of firm, rounded lobes and remains unchanged throughout diapause. If the pupa does not enter diapause, the rounded lobes disappear within 2 days^15^,^49^. Histolysis in the fat body coincides with the movement of the eye spots and is therefore a valid supplementary criterion. Because the eye spots in some pupae can be very faint, it is useful to verify the condition of the fat body in order to determine if pupae have entered diapause.

### Effects of high temperatures on the incidence of summer diapause and determination of a diapause-sensitive stage

When *H. assulta* larva is reared on the artificial diet^39^, larvae directly enter the sixth instar (final instar) from the fourth instar. In our experiment, we exposed 3^rd^-, 4^th^-, 6^th^-instar and prepupal larvae to high temperatures (33 and 35 °C) with LD 16:8 until pupation to induce diapause; we then measured the incidence of summer diapause and established which stage was sensitive to diapause induction. First, larvae were reared at 27 ± 0.5 °C and LD16:8 until the 3^rd^ instar and then reared individually to prevent cannibalism. When larvae were at the 3^rd^, 4^th^, and 6^th^ instar and the prepupal stage, they were transferred into the chambers of 33 °C with a photoperiod of LD16:8 and the chambers of 35 °C with a photoperiod of LD16:8, respectively, to induce summer diapause (80 larvae per replicate and five replicates). Prepupae were allowed to pupate in tube, and pupae were sexed and checked daily following the second day after pupation to determine if they were in summer diapause. The eye spots and round fat body remained 5 days at 33 °C/35 °C were considered to be evidence of diapause. Diapause percentage was computed as the number of diapausing pupae/the number of pupated individuals. The incidence of diapause among males and females at the 3^rd^, 4^th^, and 6^th^ instars and the prepupal stage was compared, and the sensitive stage for diapause induction was determined. At the same time, mortality was recorded and analyzed between temperature and stage exposed.

### Comparison of retention time of eye spots under temperatures maintaining and terminating diapause

To verify the occurrence of true summer diapause rather than “hot quiescence” or “hot thermal diapause”, we induce summer diapause in *H. assulta* at high temperature (35 °C) with LD16:8, non-diapausing at 27 °C with LD16:8 as control, to compare development between diapausing and non-diapausing pupae.

Larvae were reared in individual glass tubes at 27 ± 0.5 °C and LD16:8 until the final instar. Final-instar larvae (n = 400) were then transferred to a temperature regime of 35 ± 0.5 °C with a photoperiod of LD16:8 to induce summer diapause. Prepupae were allowed to pupate in tubes, and pupae were checked daily following the second day after pupation to determine if they were in summer diapause. Summer-diapausing pupae (n = 30) were maintained at high temperature (35 °C) to record the movement of eye spots to measure the duration of summer diapause. Furthermore, summer-dapausing pupae (n = 30) were kept at 35 °C for 5 days and then moved to 30 °C to terminate diapause; we measured diapause development after the transfer by recording the retention time of eye spots (The retention time of eye spots of non-diapausing pupae – n = 67 – was recorded, too.). In addition, the remaining of diapausing and non-diapausing pupae kept at 35 °C were used for following physiological measurements: body mass and mass loss, lipid and glycogen measurement, and measurement of respiratory rate.

Non-diapausing pupae (n = 20) from larvae reared at 27 °C and LD16:8 were cultured, and the retention time of eye spots was recorded (as control).

### Physiological characteristics of summer diapausing pupa of *H. assulta*

The physiological characteristics of non-diapausing pupae reared at 35 °C were compared to those of summer-diapausing pupae – body mass, water content, energy storage capacity (lipid and glycogen), and metabolism level (mass loss and respiration rate) – to evaluate the insects’ diapause preparedness and ecological adaptation to high temperature in summer.

### Body mass and water content

The diapausing pupae (n = 107) and non-diapausing pupae (n = 118) reared at 35 °C mentioned above were weighed with a balance (Sartorius Research, 0.1mg). 28 each of diapausing and non-diapausing pupa were randomly transferred to 45 °C to dry; and dry mass was weighed. Water content was calculated with the formula, (M_f_ − M_d_)/M_f_*100, where M_f_ is the fresh mass and M_d_ is the dry mass.

### Lipid and glycogen levels

On the day when summer-diapausing and non-diapausing pupae reared at 35 °C mentioned above were determined, the energy storage capacity and lipid and glycogen levels were measured. The following methods of measuring lipid and glycogen levels were used:

Weighed fresh pupae (20 each for diapausing and non-diapausing pupae) were dried to constant mass at 45 °C and then dried pupae were weighed. Dried pupae were homogenized, and their lipid content was extracted with a chloroform-methanol (2:1) solution^51^. After centrifugation (2600 g for 10 min), the supernatant was removed. The procedure was repeated twice. The resulting pellet was dried at 60 °C for 72 h and the lean dry mass (LM_d_) determined. Total lipid levels (mg/gM_d_) and lipid mass (mg) per individual were calculated using the formula: [(M_d_ − LM_d_)/M_d_] × 1000 and M_d_ –LM_d_.

Weighed fresh pupae (6 each for diapausing and non-diapausing pupae) were homogenized in 2 ml of 70% ethanol and centrifuged (2600 g for 10 min). Pooled supernatants from two replications of this procedure were discarded, and the remaining pellet was used for isolating glycogen according to the method described in Ohtsu *et al.*^52^. Two ml of 10% (v/v) trichloroacetic acid was added to the residue. The mixture was boiled in a water bath for 15 min, and then cooled and centrifuged at 3000 g for 15 min. The supernatant was used for glycogen measurement. Glycogen content was determined by the phenol and sulfuric acid method^53^. The absorbance was determined at 490 nm on a spectrophotometer (DU650, Beckman, USA). The results were expressed in mg glycogen g^−1^dw and mg glycogen/individual using a calibration curve obtained by measuring glycogen standards in seven concentrations ranging from 0.025 to 0.5 mg ml^−1^.

### Mass loss curves of body mass

Pupae were sampled from final-instar larvae reared at high temperatures (35 °C) mentioned above. Beginning on the second day after pupation, the summer-diapausing pupae (n = 61) and non-diapausing pupae (n = 48) were kept at 35 °C and weighed every other daily with a balance (Sartorius Research, 0.1 mg) – until the emergence of non-diapausing pupae – to monitor body mass changes. Mass loss per day was calculated with the formula, Δ_i_ = (M_f i_ − M_f i+2_)/2, where i is the pupal age (d) and M_f_ is the fresh mass (mg).

### Respiration rate

Respiration rate was measured to compare the differences in metabolism levels between summer-diapausing and non-diapausing pupae. The first respiration measurements were conducted at 4 pm on the second day after pupation. From then on, measurements were taken every other day at 4 pm until the emergence of non-diapausing pupae.

CO_2_ production was measured using constant volume respirometry as an indicator of overall metabolic activity. As in the method described by Sgolastra *et al.*^54^, we used a Sable Systems CA-10a CO_2_ Analyzer operating in differential mode with a 100 ml/min flow rate (http://www.sablesys.com/index.php). This allowed for an accuracy of measurement that exceeded 0.001% in detecting departures from an undepleted air stream that had been scrubbed of CO_2_ and water vapor with a Drierite1 - Ascarite1 column. At each sample date, we measured the CO_2_ produced by placing pupae individually in respiratory tubes (20 ml) for 10 min (each first run was discarded) in darkness in a 35 °C – chamber (50 replicates for each diapausing and non-diapausing pupa). Data were collected via the Sable Systems data acquisition program DATACAN1 following the manufacturer’s protocol. Upon completion of a respirometry session, individual pupae were weighed. For comparison purposes, CO_2_ levels were adjusted for the mass of each individual and expressed as 10^−4^ mol/kg/min.

### Statistical analysis

Software SPSS was used to analyzed data^55^. Statistical analyses of diapause incidence among temperatures and stages treated with high temperatures were conducted using the generalized linear model with the link function of Logit; temperatures, stages, sex and replicate were the fixed factors, considering temperature*stage, temperature*sex, stage*sex, and temperature*stage*sex. At each temperature, the generalized linear model with the link function of Logit was used to analyze the diapause incidence with stage, sex and replicate as fixed factors, considering stage*sex. For the mortality data, the generalized linear model with the link function of Logit was used with temperature, stage and replicate as fixed factors, considering temperature*stage. The general linear model was used to analyze body mass, water content, lipid, and glycogen with diapause and sex as fixed factors. Statistical analyses of data for retention time was analyzed with the generalized linear model with the link function of Poisson. Statistical analyses of data for pupal mass, and amount of body water, lipid and glycogen levels of pupae between diapausing and non-diapausing pupae were performed by one-way analysis of variance (ANOVA), and the means were separated with the Scheffe’s multiple range test. Statistical analyses of body mass changes and respiration rate changes among pupal age were conducted by the general linear model with repeated measurements so that pupal age and diapause were within-subjects variable and between-subjects factor, respectively, and the means were separated with the Scheffe’s multiple range test.

## Additional Information

**How to cite this article**: Liu, Z. *et al.* Summer diapause induced by high temperatures in the oriental tobacco budworm: ecological adaptation to hot summers. *Sci. Rep.* **6**, 27443; doi: 10.1038/srep27443 (2016).

## Supplementary Material

Supplementary Figure S1

## Figures and Tables

**Table 1:** Comparison of retention times of pigmented eye spots.

Pupae	n	Retention of pigmented eye spots (day)
**(A)** Among summer diapausing, non-diapausing pupae reared at 35 °C and non-diapausing pupae reared at 27 °C		
Summer-diapausing pupae reared at 35 °C	30	13.43 ± 1.04a
Non-diapausing pupae reared at 35 °C	67	1.76 ± 0.12b
Non-diapausing pupae reared at 27 °C	20	1.58 ± 0.10b
Wald Chi-Square		293.759
df		2
P		<0.001
	**Retention of pigmented eye spots (day)**	
	**n**	**Storage**^a^
**(B)** Under diapausing termination condition		
Summer-diapausing induced by high temperature	30	8.45 ± 0.81a
Non-diapausing pupae reared at 35 °C	67	1.76 ± 0.12b
Non-diapausing pupae reared at 27 °C	20	1.58 ± 0.14b
Wald Chi-Square		145.842
df		2
P		<0.001

The data in the table are Mean ± SE and those followed by different letters differ significantly by the generalized linear model (P < 0.05).

^a^Before being transferred to conditions of 27 °C, summer-diapausing pupae were kept at 35 °C for 5 day.

**Table 2:** Body mass and energy storage capacity of summer-diapausing and non-diapausing pupae experiencing the high temperature 35 °C.

	Non-diapausing pupae	Summer-diapausing pupae	F	p
Fresh body weight (mg)^a^	247.2 ± 4.4 (118)	268.0 ± 3.9 (107)	12.231	0.01
Water content (% DW)^b^	63.2 ± 1.4 (28)	63.5 ± 0.9 (28)	0.637	0.428
Lipid				
mg/pupa	40.3 ± 1.8 (21)	48.5 ± 1.9 (21)	9.992	0.003
mg/g DW	548.6 ± 11.2 (21)	551.1 ± 7.6 (21)	0.033	0.857
Glycogen				
mg/pupa	2.3 ± 0.4 (6)	4.1 ± 0.5 (6)	7.865	0.019
mg/g DW	27.0 ± 3.5 (6)	43.5 ± 5.0 (6)	7.33	0.022

The data in the table are Mean ± SE and the values in parenthesis are the number of samples. The data of percentages were arcsine-transformed before analysis and the untransformed data are shown in the table.
