# Supplementary material for: Summer diapause induced by high temperatures in the oriental tobacco budworm: ecological adaptation to hot summers
Source: Sci Rep. 2016 Jun 7;6:27443. doi: 10.1038/srep27443 (PMC4895216; doi:10.1038/srep27443)

Classification: Biological Sciences: Ecology

Summer diapause induced by high temperatures in the oriental tobacco budworm: ecological adaptation to hot summers

Zhudong Liu[a, *], Yucui Xin[a, b], Zhang Yanan[a,c], Fan Jianting[c], Sun Jianghua[a, *],

[a]The State Key Laboratory of Integrated Management of Pest Insects and Rodents, Institute of Zoology, Chinese Academy of Sciences, Beijing 100080.

[b]Institute of Health Sciences, Anhui University, Hefei 230039, China.

[c]College of Forests and Biology, Agriculture and Forest University of Zhejiang, Hangzhou 310000, China.

[*]To whom correspondence should be addressed. Email: liuzd@ioz.ac.cn and sunjh@ioz.ac.cn

Keywords: summer diapause, *Helicoverpa assulta*, high temperatures, body mass and energy storage capacity, metabolism

Supplementary information

Fig. S1 Eye spots and fat body of developing pupa reared at 27 ℃. A and B, showing the presence of eye spots and round fat body with blue arrow marker when it is just pupated, respectively; C and D, showing the disappearance of eye spots and round fat body, respectively.

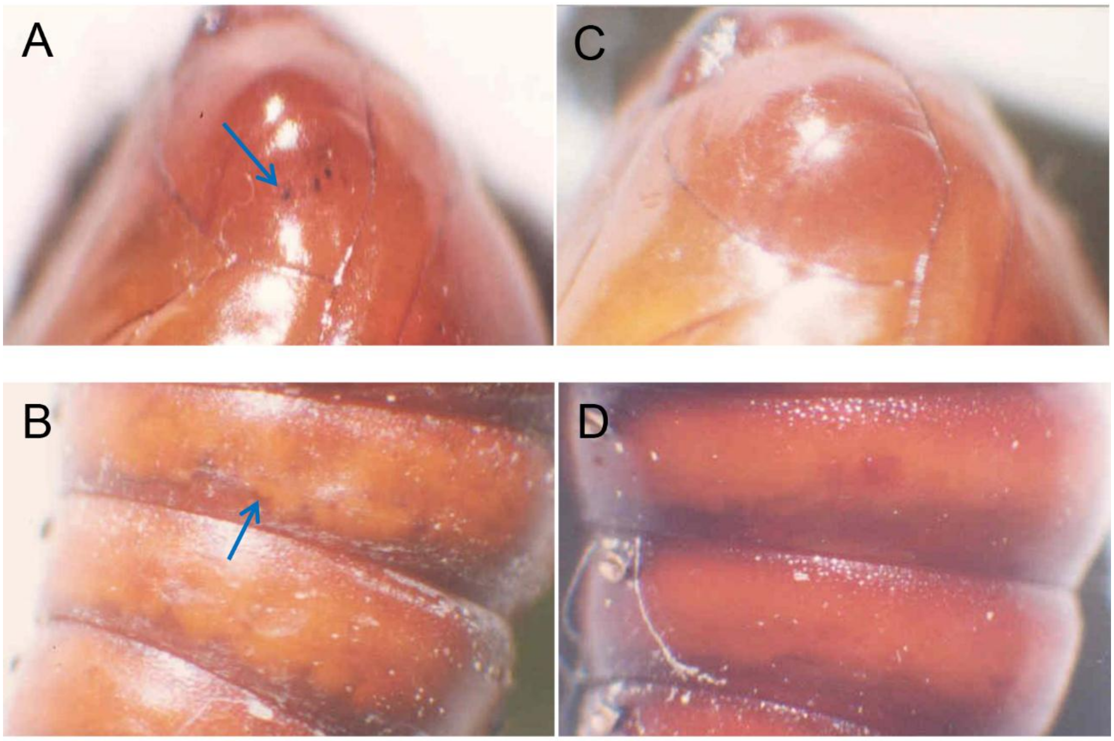

Supplement: Supplementary Figure S1 [file srep27443-s1.pdf]
